# Mechanisms of Movement Patterns: Physiology and Sex Influence Gopher Tortoise (*Gopherus polyphemus*) Movement

**DOI:** 10.1002/ece3.73878

**Published:** 2026-06-30

**Authors:** Karin Ebey, Michael Hilton, Jeffrey Goessling

**Affiliations:** ^1^ Eckerd College Natural Sciences Collegium St. Petersburg Florida USA; ^2^ Odum School of Ecology, University of Georgia Athens Georgia USA

**Keywords:** gopher tortoise, GPS logger, movement ecology, physiological constraint

## Abstract

A central topic in spatial ecology is identifying what determines animal movement patterns, including both exogenous and endogenous factors. Given that there are different sex‐based selection pressures in mating and reproduction, movement patterns often vary between males and females in predictable manners; males of polygynous species typically move more than females to maximize mating opportunities. In female polygynous animals, selection is generally thought to favor movement patterns with smaller home ranges and high spatial site fidelity. Physiology may also constrain movement patterns in a way that can obscure sex‐specific selection. We used GPS loggers and physiological assays to study Gopher Tortoise ( *Gopherus polyphemus* ) movement patterns to determine how movement patterns depend on physiology (as measured by the baseline and the change in plasma lactate concentration in response to standardized exercise challenge and body condition) and movement strategy (total distance traveled over the course of the study, daily distance traveled, maximum daily displacement, home range area, burrow use) by sex. Total distance traveled was positively related to body condition and negatively related to baseline lactate concentration (which is an inverse metric of aerobic fitness). Thus, total distance traveled was physiologically constrained. Change in plasma lactate concentration, however, was not related to any of the space usage parameters. Home range area and burrow use were not related to any physiological metrics. There was not a significant difference in total distance traveled nor daily distance traveled between male and female Gopher Tortoises, but males had larger maximum daily displacements than females. Males also had larger home range areas. Male and female Gopher Tortoises thus used different movement strategies that may have maximized fitness consistent with different selection pressures by sex in a scramble competition mating system.

## Graphical abstract

This study tested physiological mechanisms of movement patterns in Gopher Tortoises (*Gopherus polyphemus*) by using autonomous GPS loggers. Total distance traveled, but not home range area, was physiologically limited. Females traveled the same distance as males, but had smaller home range areas, which challenges traditional understanding of sex‐based movement tradeoffs and spatial ecology.

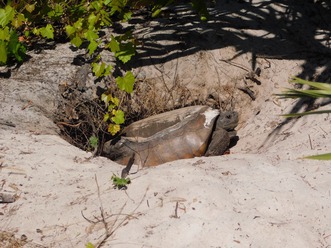

## 1 Introduction

One of the defining features of animals is their ability to move. Through movement, animals are able to interact with other organisms, such as prey, predators, and conspecific individuals (McLoughlin and Ferguson 2000; Nathan et al. 2008; Wu and Seebacher 2022). Thus movement patterns influence an individual's ecological interactions and in turn its evolutionary fitness through effects on survival and reproduction rates. A central question in spatial ecology is identifying what context drives specific movement patterns or the decisions that animals make in moving. Animal movement patterns are dependent on both exogenous factors such as food availability and population density, and endogenous factors such as the ability to move and motivation for movement (Hetem et al. 2025; McLoughlin and Ferguson 2000; Nathan et al. 2008). Here we focus on internal factors driving animal movement patterns in a social vertebrate, *Gopherus polyphemus* , the Gopher Tortoise.

Physiological traits, such as endurance, condition, metabolic rate, and cardiovascular output, influence animal movement patterns (Hetem et al. 2025). For example, home range area in Desert Iguanas ( *Dipsosaurus dorsalis* ) increases with aerobic endurance (Singleton and Garland 2018). In Bank Voles ( *Myodes glareolus* ) movement displacement and home range area increase with aerobic capacity (Boratyński et al. 2020). Across many taxa (vertebrates and invertebrates), a meta‐analysis found significant positive relationships between physiological condition, metabolic rate, and locomotor capacity and amount of movement (Wu and Seebacher 2022).

Animals move for a variety of reasons such as mate or prey seeking, territory defense, and predator avoidance, but ultimately movement is limited due to time and physiological constraints. Movement patterns and strategies vary by sex in a variety of taxa likely in response to sex‐specific selection pressures, due to differential reproductive investment and the mating system. For example, in Magnificent Frigatebirds ( *Fregata magnificens* ), males and females have similar foraging effort, but females do not displace as far as males because they remain close by to provide extensive care to offspring (Austin et al. 2019). In Bobcats ( *Lynx rufus* ) and Eurasian Lynx ( *Lynx lynx* ), males have larger home range areas than females to maximize mating opportunities and ensure access to females (Aronsson et al. 2016; Ferguson et al. 2009). Sex‐specific movement patterns can also vary seasonally depending on requirements for reproduction. For example, male Sleepy Lizards (*Tiliqua rugosa*) travel extensively to find mates during the mating season, whereas female Sleepy Lizards move more after the mating season to forage for sufficient energy for reproduction (Kerr and Bull 2006); male Yellow‐Bellied Slider Turtles ( *Trachemys scripta* ) move more throughout the year, except during nesting season when females search for nest sites (Morreale et al. 1984).

The Gopher Tortoise, *Gopherus polyphemus* , is a threatened keystone species that constructs and inhabits burrows in upland habitats of the southeastern United States (Catano and Stout 2015). *Gopherus polyphemus* exhibits social behaviors, living in groups and forming social networks (Guyer et al. 2014; Hilton et al. 2024). The mating system of *Gopherus polyphemus* is complex (Johnson et al. 2007; Moon et al. 2006; Tuberville et al. 2011; White et al. 2018; Yuan et al. 2019), and there is growing consensus that the mating system involves scramble‐competition polygyny, where individual males have equal odds of mating once they find a receptive female (Boglioli et al. 2003; Johnson et al. 2009; White et al. 2018). However, there is some evidence for female‐defense polygyny, where males guard females they mate with and competitively superior males have greater chances of mating (Johnson et al. 2009; Tuberville et al. 2011; Guyer et al. 2014; White et al. 2018). Between *Gopherus polyphemus* populations, movement varies with population density, peaking at intermediate population densities likely balancing trade‐offs between energy expenditure and ensuring access to conspecifics (Guyer et al. 2012). Movement generally peaks during the mating season (Eubanks et al. 2003). Prior studies of *Gopherus polyphemus* movements have found that males have larger home ranges and displacement distances than females as a result of traveling to mate (Castellón et al. 2018; Eubanks et al. 2003; Guyer et al. 2012; McRae et al. 1981; Metcalf et al. 2023), but see Guyer et al. (2024). At the individual scale, home range size is also related to physiology with a negative correlation between baseline lactate and corticosterone levels and home range size, implying that individuals with greater aerobic fitness and lower stress levels use more area (Stiffler et al. 2024).

Most previous studies of tortoise movement patterns used radiotelemetry or direct observation, with coarse temporal resolution, 1–3 locations per week (Castellón et al. 2018; Douglass and Layne 1978; Guyer et al. 2012; Stiffler et al. 2024). The field of spatial ecology is currently undergoing a revolution with the increasing accessibility of GPS logger technology, which autonomously collects locations at much higher frequencies, with less effort and disturbance, relative to radiotelemetry or direct observations (Calabrese et al. 2016; Crane et al. 2021; Fleming et al. 2015; Hetem et al. 2025; Nathan et al. 2008; Silva et al. 2022). These new technologies allow a more nuanced view of animal movement patterns because temporally fine‐scale location data facilitate accurate determination of metrics like total linear distance traveled and number of times specific locations are visited (Nathan et al. 2022). Many studies generalize animal movement with home range area because this metric can be reasonably calculated with location data on the order of 10–100 points per individual (Crane et al. 2021). However, animal movement patterns ultimately are the synthesis of the path followed by individuals, and so should be characterized by multiple metrics such as home range area, total distance traveled, and locations visited depending on the questions of interest (Nathan et al. 2022). The use of multiple metrics may clarify what individual animals do within their movement budget, such as assessing the relative amount of short versus long distance movements or determining if certain locations are visited repeatedly. Relationships between movement metrics or lack thereof have been noted in various taxa. For example, in Wild Boars **(* Sus scrofa)* there was not a strong relationship between monthly distance traveled and home range area (Cavazza et al. 2024). Whereas home range area in Pine Snakes ( *Pituophis melanoleucus* ) increased with distance traveled from hibernaculum sites (Zappalorti et al. 2015), and in rodents ( *Akodon montensis* and *Delomys sublineatus* ) home range area was correlated with linear distance traveled between captures (Püttker et al. 2012). Ultimately, this illustrates the need for data capturing multiple movement metrics to understand the nuances of movement patterns because they cannot be assumed to be linearly related.

Our goal was to test connections between movement patterns and individual anatomy and physiology to identify constraints and drivers of movement patterns in a social habitat specialist, *Gopherus polyphemus*, the Gopher Tortoise. Our preliminary question was: How are the different movement metrics of home range area, total distance traveled, daily distance traveled, daily maximum displacement, number of burrows visited, and number of burrows occupied related to each other? We hypothesized that these movement metrics would be positively related to one another because a larger home range would contain more burrows and require traveling greater distances to cover the home range. This first question was one largely of methodological improvement and validation. Our subsequent and driving research question was: How does movement, as measured by total distance traveled, daily distance traveled, daily maximum displacement, home range area, and burrow use (number visited and number occupied), vary with size, body condition index, and aerobic physiology, as measured by change in lactate, and sex? We hypothesized that individuals with higher body condition and greater aerobic fitness, as assessed by a smaller change in lactate following exercise, would travel farther, have a larger home range, and use more burrows. Based on the literature, we hypothesized that males would travel farther, have a larger home range and daily maximum displacement, and use more burrows than females because of selection to increase mating opportunities.

## 2 Methods

### 2.1 Study Site

This study took place at Boyd Hill Nature Preserve in St. Petersburg, Florida, USA, about 100 ha in size (see map in Figure 2). We focused on the *Gopherus polyphemus* population that lives in the southeastern corner of the preserve which is approximately 40 ha of open‐canopy scrubby flatwoods and well‐drained very fine sandy soil, managed with prescribed fires every 1–3 years. The habitat is dominated by Wiregrass ( *Aristida beyrichiana* ), Longleaf Pine ( *Pinus palustris* ), South Florida Slash Pine (*Pinus elliottii densa*), Saw Palmetto ( *Serenoa repens* ), abbage Palm ( *Sabal palmetto* ), Sand Live Oak ( *Quercus geminata* ), Myrtle Oak ( *Q. myrtifolia* ), Chapman's Oak (*Q. chapmanni*), Sand Laurel Oak ( *Q. hemisphaerica* ), and Virginia Live Oak ( *Q. virginiana* ). The study site has an elevation of ~2 m above sea‐level and a subtropical climate with average annual temperature of 24.4°C and wet‐dry seasonality with May–October as wet season and November–April as dry season. The *Gopherus polyphemus* population in Boyd Hill Nature Preserve is estimated to be around 100 adults and thus has a high population density (Goessling et al. 2025).

### 2.2 Field Methods

Between 12 August 2024 and 21 September 2024, we hand captured 30 adult *Gopherus polyphemus* , all of which had Carapace Length at least 24 cm (Landers et al. 1982; McRae et al. 1981), at Boyd Hill Nature Preserve in St. Petersburg, Florida. The date, time, and location of each tortoise was recorded. We also noted whether the tortoise was caught in their burrow or out of their burrow actively walking around. The sex of each tortoise was identified by presence or absence of the male secondary sex traits of enlarged gular projection and plastron concavity. The gular projection is used as a weapon in male–male competition, and its length is sexually dimorphic (Guyer et al. 2014). Immediately upon capture, we tested the tortoise's aerobic performance by measuring change in lactate concentration in response to exercise (Hawthorne and Goessling 2020). Lactate levels have been used as an indicator of exercise performance where a smaller increase in lactate in response to exercise indicated that an animal has a greater capacity for aerobic respiration and relies less on anaerobic respiration (Gardner et al. 2022; Hawthorne and Goessling 2020; Tracy et al. 2012). Using a 25‐gage needle, we pricked a front foot for a drop of blood to measure the baseline blood lactate concentration with a Lactate Plus lactate meter (Nova Biomedical) (Vergneau‐Grosset et al. 2025). The tortoise's temperature was measured with an instant‐read digital thermometer inserted ~2 cm into the cloaca. Then the tortoise walked for 400 paces, defined as a single forward step by one (right or left) forelimb, on a custom‐made, hand‐powered treadmill as shown in Figure S1, noting the time to complete the challenge. We aimed for every tortoise to walk at the same pace, about 1 revolution per second on the hand crank. If tortoises were not moving at that pace, we gently prodded the posterior carapace to stimulate them to step forward. Lactate concentration and temperature were measured postexercise as before. We chose 400 paces for the physiological challenge because based on preliminary observations tortoises were still willing to walk, and in a preliminary trial blood lactate concentration increased from 3.4 mM to 5.6 mM. We limited the total number of paces to 400 because in preliminary trials we found that many more paces caused tortoises to stop walking, thereby confounding behavior with lactic acid accumulation (Adamovicz et al. 2018; Klein et al. 2021). The time spent walking was not limited, but was noted, and ranged between 260 and 670 s.

Using calipers, we measured the straight midline carapace (CL) and gular length (GL) of each tortoise. Gular length is suggested as an indicator of male dominance (Guyer et al. 2014) and so was included in the analyses of movement patterns. Using a digital scale, we measured the mass of each tortoise. We attached a W510 GPS Logger (Advanced Telemetry Systems) to the right posterior of the carapace using epoxy (J.B. Weld). The GPS loggers were 65 g and the combined weight of a logger and adhesive epoxy was less than 5% of each tortoise's mass. The loggers were programmed to record GPS location every 15 min from 0700 to 2100 h. As GPS location fixes were not likely when the tortoise was underground in the burrow due to interference of the substrate (Nowakowski et al. 2020), we programmed the loggers to only attempt 2 fixes per assigned time to prolong the battery life. We remotely downloaded the data from the GPS loggers approximately once a month and at the conclusion of the study period using W100 Remote Comm Module and Wildlink High Range Yagi (Advanced Telemetry Systems). Locations were collected through 25 February 2025. Gopher Tortoises nest once per year during the late spring and early summer (Allman et al. 2019; Ott et al. 2000), and in peninsular Florida, where this study took place, Gopher Tortoises exhibit courtship and mating behaviors year‐round, peaking outside of the nesting season (Allman et al. 2019; Hilton 2023). Thus, all locations recorded during this study fell outside of the nesting season.

### 2.3 Analyses

All analyses were conducted in R version 4.3.2 (R Core Team 2023) and the packages ggplot2 and leaflet were used for data visualization (Cheng et al. 2024; Wickham 2016). We used 0.05 as the threshold for significance. Unless otherwise noted, significant outliers were detected using the identify_outliers function of the rstatix package (version 0.7.2; Kassambara 2023), with outliers being 1.5× the interquartile range and extreme outliers being 3× the interquartile range.

The change in lactate concentration was calculated as the difference between the final and initial (pre‐ and postexercise) lactate concentration for each tortoise. Body condition index (BCI) was calculated separately for males and females by finding the standardized residuals from the line of best fit of log‐transformed mass versus log‐transformed carapace length (Polo‐Cavia et al. 2010). Body condition was plotted as a function of carapace length, and the *p*‐value of a linear model was used to test for a relationship between body condition and carapace length, to ensure that body condition was independent of size.

This study focused on location data collected during the 158 day period between 21 September 2024 and 25 February 2025, when all individuals in the study had functioning GPS loggers. The simplest method for calculating the distance between consecutive GPS locations is to use the Euclidean distance, which assumes straight‐line motion. Our field observations at Boyd Hill indicated tortoises did not typically move in straight lines; instead, they constantly adjusted their direction of travel to navigate through local landscape features. Therefore, we chose to use a continuous‐time movement model implemented by the ctmm package (version 1.2.0; Calabrese et al. 2016) to incorporate such motions into our distance calculations. In addition, the movement and home range models in the ctmm package were designed to deal with GPS location errors and spatial autocorrelation (Averill‐Murray et al. 2020; Calabrese et al. 2016; Fleming et al. 2017, 2015; Silva et al. 2022). The movement data were prepared for analysis using the calibration methodology described in Fleming et al. (2020), which is supported by the ctmm package. A calibration dataset of 110 location fixes from a stationary GPS logger were collected to determine the best model for User Equivalent Range Error (UERE). The ctmm::uere.fit function was used to fit candidate error models incorporating information provided by the GPS logger for each successful location fix, specifically: horizontal dilution of precision (HDOP), number of satellites, and length of time required to obtain a fix. Additionally, a null model assuming all GPS locations have the same variance rather than varying based on signal quality was fit. All these models were compared using Akaike Information Criterion corrected for small samples sizes (AICc) to select the most informative calibration model (Fleming et al. 2020), which was then applied to the tortoise location data. We then used the ctmm::outlie function to identify location outliers (0.09% of the data points) for removal based on each tortoise's location and speed of travel (Calabrese et al. 2016). A continuous‐time movement model was fit for each tortoise using the ctmm::select function, and this model was used by the ctmm::distances and ctmm::akde functions in the calculation of distance between GPS locations and home ranges, respectively.

Tortoise movement was described using six metrics which reveal different aspects of space use: distance traveled each day; maximum displacement from the first location recorded of the day, which was taken as a proxy for the location of the burrow currently occupied by the tortoise; total distance traveled over the entire duration of the study period; home range area, as measured by 95% AKDE; number of burrows visited over the duration of the study period; and number of burrows occupied during the study period.

The daily distance traveled was calculated by summing the travel distance between consecutive GPS fixes taken on the same day, as determined by the ctmm::distances function, and the long‐term total distance traveled was calculated by summing the daily distances. The daily maximum displacement was calculated as the maximum Euclidean distance of each GPS location from the first GPS location of the day. We calculated home range for the duration of the study period using 95% autocorrelated kernel density estimate (AKDE) computed by the ctmm:akde function. We determined if a tortoise was home range resident by creating a variogram, and checking for an asymptote as time lag increases in the variogram (Calabrese et al. 2016). The home range area for two tortoises (a male: UNM‐1 and a female: 311) were identified as outliers, and variogram analysis indicated these tortoises were not home range resident. As the home range areas for UNM‐1 and 311 were high leverage values and distorted correlation tests and linear regressions, they were excluded from analyses involving home range area (i.e., summary statistics of home range area by sex, correlations between movement metrics, and linear model relating physiology and anatomy to home range area as described below). To determine burrow use, first we identified candidate burrow locations as places where a tortoise was stationary, defined as less than 5 m of motion between radio fixes, for at least 1 h (Douglass and Layne 1978) within the tortoise movement data. Burrow locations were identified by clustering the candidate locations using hierarchical agglomerative clustering with the hclust and cutree functions in R (R Core Team 2023) assuming a maximum of 120 burrows and only including locations that were visited at least three times over the course of the study. We determined the total number of burrows a tortoise visited as the number of unique burrows its location points came within 5 m of, using buffer and intersect tools in ArcGIS Pro (ESRI 2024). We determined the total number of burrows that each tortoise occupied by the number of unique burrows that the tortoise were within 5 m of at the start of each day, because tortoises start their day basking at the burrow they occupied overnight (Douglass and Layne 1978).

A Pearson correlation matrix was computed for each sex to assess the relationship between each pair of movement metrics. The matrix was visualized using the packages corrplot (version 0.95; Wei and Simko 2024) and Hmisc (Harrell Jr 2025). To include the daily distances traveled and daily maximum displacements in the correlation tests, it was necessary to reduce them to single values per tortoise by taking their means.

To test the hypothesis that males travel longer distances than females, we compared both long‐term and short‐term movement patterns. The number of males and females were not balanced, so a one‐sided Welch's two sample *t*‐test was used to test the alternative hypothesis that males traveled further than females. The short‐term movement was tested using the daily distance traveled by each tortoise and the daily maximum displacement for each tortoise. The unbalanced count of males and females rules out the use of repeated‐measures ANOVA for analysis; a linear mixed‐effects model was more appropriate, with sex, day, and sex:day interaction treated as fixed effects, and subject treated as a random effect with each subject (i.e., tortoise) having a separate intercept. The decision not to include separate slopes for the random effect was based on visual inspection of the data, which suggested the slope was close to zero for all tortoises. This model was expressed as:

$$\text{measure}\sim \mathrm{sex}+\mathrm{day}+{\mathrm{sex}}^{*}\;\mathrm{day}+\left(1\;|\;\text{subject}\right)$$

where measure was either daily distance traveled or daily maximum displacement. The null hypothesis for each measure was ${\text{measure}}_{\text{male}}={\text{measure}}_{\text{female}}$; the alternative hypothesis was ${\text{measure}}_{\text{male}}\neq {\text{measure}}_{\text{female}}$. Visual inspection of daily measures indicated that every tortoise had multiple days in which they did not leave their burrow, resulting in nonnormal daily measures with outliers, necessitating the use of a linear mixed‐effects model implementation with a robust estimation method. We used the rlmer function of the robustlmm package (version 3.3–2; Koller 2016). The rlmer function computes a *t*‐value for each fixed effect, but there is no widely agreed upon way to generate *p*‐values for the fixed effects due to difficulties in computing degrees of freedom. An alternative approach to inference testing is to use bootstrapping to compute a confidence interval for the *t*‐value. If the confidence interval includes zero, the alternative hypothesis is not supported. The confintROB function of the confintROB package (version 1.0–1; Mason et al. 2024) was used to compute confidence intervals for inference testing.

Using data only from tortoises with lactate measurements, we tested whether home range area (95% AKDE), total distance traveled, and burrow use (number visited and number occupied) varied with tortoise sex, CL, GL, mass, BCI, initial lactate concentration, and change in lactate concentration using multiple linear regression. For each multiple linear regression, an ANOVA was run on the complete model to identify predictor variables with a significant association to the dependent variable. These significant predictor variables were included in submodels. All models were assessed via AICc to identify the most informative model using the package AICcmodavg (version 2.3–4; Mazerolle 2023). A correlation matrix was created by calculating the Pearson correlation and significance of the relationship between each pair of variables of tortoise CL, GL, mass, BCI, initial lactate, change in lactate, and total distance traveled, 95% AKDE, and number of burrows visited and occupied.

We tested whether initial lactate concentration varied with the tortoise activity (out/active or in burrow), temperature, sex, CL, GL, and BCI using multiple linear regression. We tested whether the change in lactate concentration varied with the tortoise activity, temperature, time spent walking the 400 paces, CL, GL, mass, and BCI. We considered activity status and temperature because tortoises are ectotherms, so their metabolism and locomotor performance depend on body temperature (Elnitsky and Claussen 2006). For multiple linear regressions, an ANOVA was run on the complete model to identify predictor variables with a significant association to the dependent variable, which were included in submodels. All models were assessed via AICc to identify the most informative model. A correlation matrix was created by calculating the Pearson correlation and significance of the relationship between each pair of variables of tortoise activity (out/active or in burrow), temperature, time spent walking the 400 paces, CL, GL, mass, BCI, initial lactate, and change in lactate.

## 3 Results

We collected data from 30 *Gopherus polyphemus* , 16 male and 14 female. We took initial lactate measurements from 26 individuals and 25 successfully completed the treadmill assay, yielding final lactate and change in lactate measurements. Thus, for the regression analyses between movement parameters and anatomy and physiology traits the dataset includes only 25 tortoises (14 male, 11 female), but analyses examining the relationships between the different movement metrics and comparing movement patterns by sex include all 30 tortoises.

The UERE model for the calibration data with the lowest AICc was the null model that assumed all GPS locations have the same error variance, so that was used to calibrate the tortoise location data. According to this model, an HDOP of 1.0 was equivalent to a location error of 5 m.

Over the 158‐day study period, between 21 September 2024 and 25 February 2025, 177,475 location points were recorded with a mean of 5916 (range: 2567–7870) positions recorded from each tortoise.

### 3.1 Movement Metrics by Sex

**FIGURE 1:**
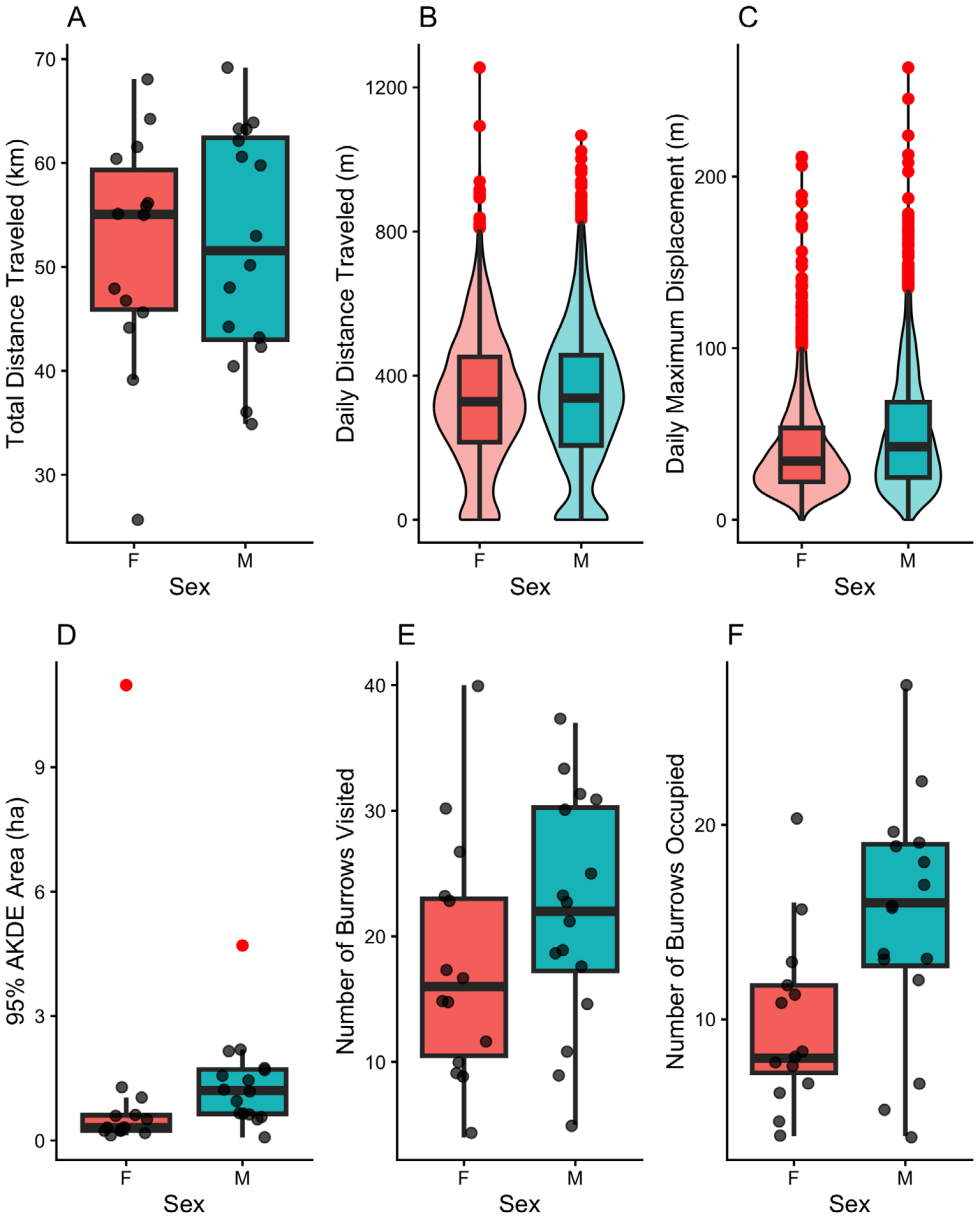
Box plots of the total distance traveled over the duration of the study (A), the daily distance traveled (B), and the daily maximum displacement from the first GPS location of the day (C), home range area as measured by 95% AKDE (autocorrelated kernel density estimate) (D), the number of burrows visited over the duration of the study period (E), and the number of burrows occupied over the duration of the study period (F). Outliers are colored red. Original data are shown as jittered points or distribution with violins.

The movement metrics for each individual are reported in Supplemental Table S1. The mean total distance traveled over the duration of the study period for males was 52,152 m ± 11,143 SD (range: 34,885 m–69,186 m; *n* = 16) and for females was 51,832 m ± 11,186 SD (range: 25,655 m–68,074 m; *n* = 14); see Figure 1A for box plots of the total distance values. No significant outliers were detected for either sex. The male total distance distribution was normally distributed (Shapiro–Wilk *W* = 0.924, *p* = 0.196). The female total distance distribution was mildly thick‐tailed (kurtosis = 3.226), but not enough to be considered nonnormal by the Shapiro–Wilk test (*W* = 0.949, *p* = 0.547). Therefore, use of the one‐sided Welch's two‐sample *t*‐test to determine if there was a sex difference in the total distance traveled is sound; this test indicated no support for the alternative hypothesis that males traveled farther than females (*t* = −0.078, df = 27.444, *p* = 0.469).

The mean of all daily distances traveled for the males was 332 m ± 197 SD (range: 0 m–1067 m; *n* = 2512) and for the females was 330 m ± 183 SD (range: 0 m–1254 m; *n* = 2198). Box plots of the daily distances are shown in Figure 1B. There were 37 significant outliers, one of which was labeled extreme; none of these outliers were high leverage values, so none were removed from the analysis. The Shapiro–Wilk test indicated neither the male nor female daily distance distributions were normal (males: *W* = 0.977, *p* < 0.001; females: *W* = 0.984, *p* < 0.001). The robust linear mixed‐model found the fixed effect of sex was not statistically significant (β = 36.855, SE = 32.019, 95% CI = [−23.155, 97.311], *t* = 1.151), indicating a lack of support for the alternative hypothesis that males and females travel different daily distances.

The mean of all daily maximum displacement from the day's first GPS location for the males was 51 m ± 34 SD (range: 0 m–263 m; *n* = 2253) and for the females was 41 m ± 27 SD (range: 0 m–212 m; *n* = 2045). Box plots of the daily maximum displacement are shown in Figure 1C. There were 156 significant outliers, 27 of which were labeled extreme; none of these outliers were high leverage values, so none were removed from the analysis. The Shapiro–Wilk test indicated neither the male nor female daily distance distributions were normal (males: *W* = 0.908, *p* < 0.001; females: *W* = 0.865, *p* < 0.001). The robust linear mixed‐model found the fixed effect of sex was statistically significant (β = 21.396, SE = 3.262, 95% CI = [11.716, 31.369], *t* = 6.559), indicating strong support for the alternative hypothesis that males and females have different daily maximum displacements. It is clear from Figure 1C that females tended to stay closer to their burrow than males do.

**FIGURE 2:**
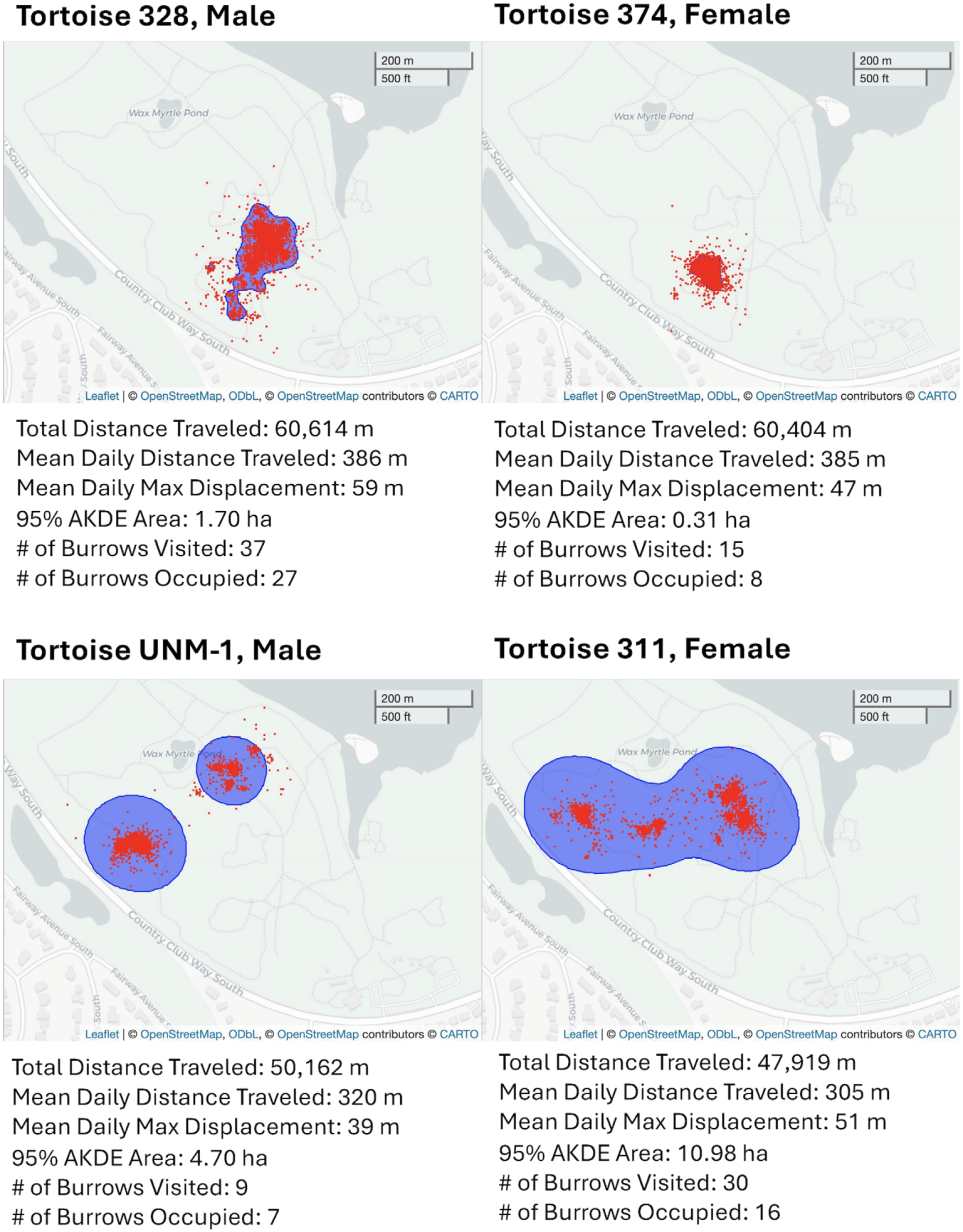
A comparison of home ranges and movement metrics for four tortoises. The home range areas of tortoises 328 and 374 are close to the median areas for males and females, respectively. Tortoises UNM‐1 and 311 occupied multiple widely spaced burrows over the course of the study and their home range areas are outliers (and so were excluded from analyses involving home range area), yet their other movement metric measurements are not outliers.

Box plots of the 95% AKDE home range areas are shown in Figure 1D. The home range area for male tortoise UNM‐1 was an outlier (4.702 ha) and female tortoise 311 was an extreme outlier (10.979 ha). A graphical comparison of the movement of these two tortoises with outlier home range area with two other tortoises having areas close to the median for each sex is presented in Figure 2. Interestingly, none of the other distance metrics for tortoises UNM‐1 and 311 were outliers. Variogram analysis (Calabrese et al. 2016) indicated these tortoises were not home range resident and from the maps in Figure 2 it is apparent that both tortoises took up residence in several widely spaced burrows over the duration of the study. The mean home range area for the remaining males was 1.150 ha ± 0.640 SD (range: 0.076 ha–2.194 ha; *n* = 15) and for the remaining females was 0.459 ha ± 0.350 SD (range: 0.126 ha–1.283 ha; *n* = 13). The home range data were not normally distributed (Shapiro–Wilk *W* = 0.899, *p* = 0.011), so a Mann–Whitney‐Wilcoxon test was used to determine that there was a significant difference between the male and female area distributions (*W* = 32, *p* = 0.002).

The mean number of burrows visited by males over the duration of the study period was 22 ± 9 SD (range: 5–37; *n* = 16) and for the females was 18 ± 10 SD (range: 4–40; *n* = 14); box plots of the visit counts are shown in Figure 1E. The number of burrows visited was normally distributed for both sexes (male: *W* = 0.974, *p* = 0.900; female: *W* = 0.945, *p* = 0.492) and a Welch Two Sample *t*‐test indicated there was no significant difference by sex (*t* = −1.134, df = 26.861, *p* = 0.264).

The mean number of burrows occupied by males over the duration of the study period was 15 ± 6 SD (range: 4–27; *n* = 16) and for the females was 10 ± 4 SD (range: 4–20; *n* = 14); box plots of the burrow counts are shown in Figure 1F. The number of burrows occupied was normally distributed for both sexes (male: *W* = 0.964, *p* = 0.742; female: *W* = 0.923, *p* = 0.245) and a Welch Two Sample *t*‐test indicated there was a significant difference by sex (*t* = −2.719, df = 26.999, *p* = 0.011).

**FIGURE 3:**
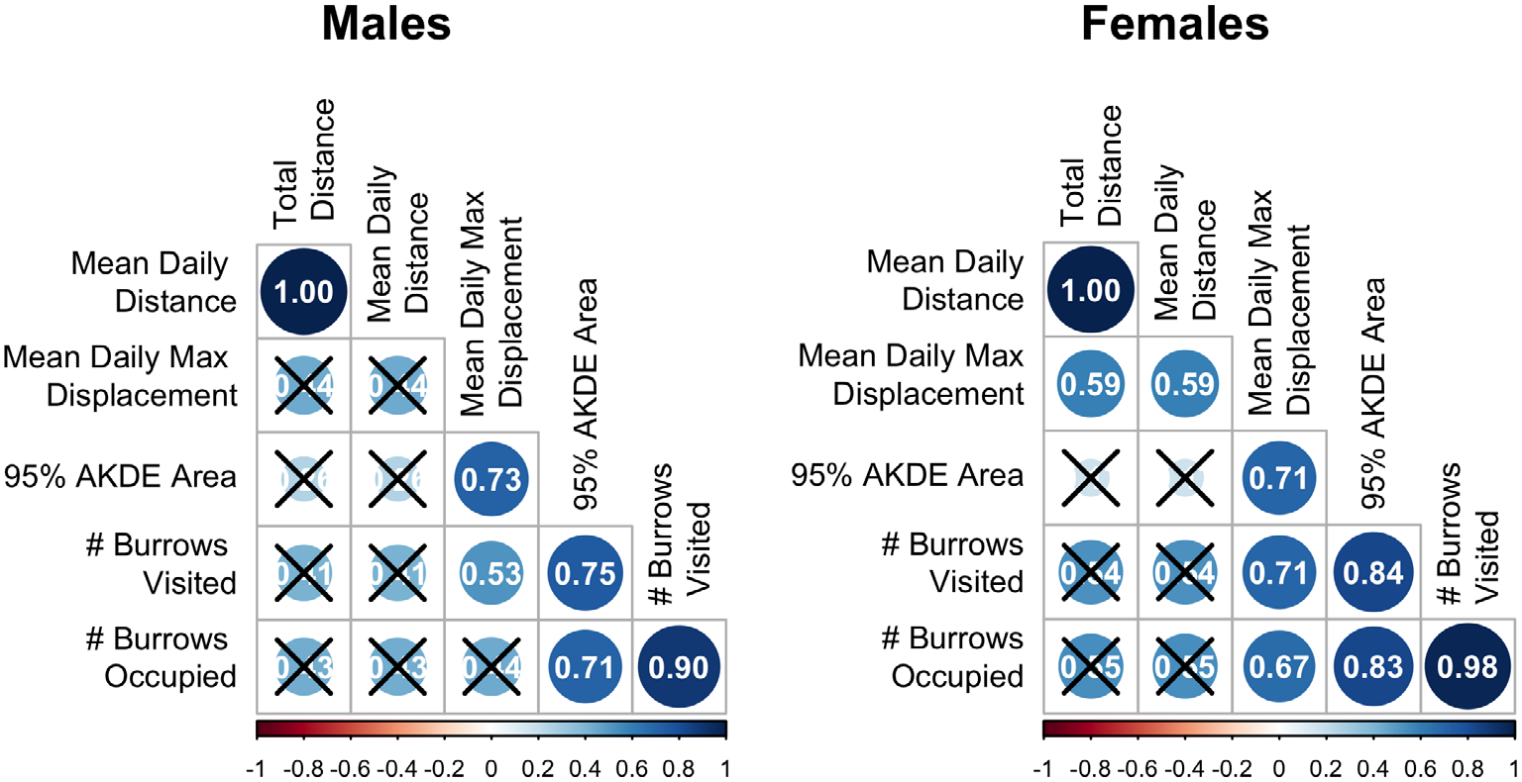
Pearson correlation matrix assessing the linear relationship between each pair of distance metrics: The total distance traveled over the duration of the study, the mean of daily distances traveled, the mean of daily maximum displacements from the first GPS location of the day, the home range area as measured by 95% AKDE (autocorrelated kernel density estimate), the number of burrows visited over the duration of the study period, and the number of burrows occupied over the duration of the study period. Circle size, color, and text indicate the correlation between each pair of variables, and an “X” indicates nonsignificant correlations.

A Pearson correlation was computed to assess the relationship between each pair of distance metrics. The results were slightly different for males (*n* = 15) and females (*n* = 13); the *r* values for significant correlations are presented in Figure 3. Home range area was positively correlated with number of burrows visited and number of burrows occupied for both sexes. The mean daily maximum displacement was positively correlated with every distance metric for the females, but only with the home range area and number of burrows visited for the males.

### 3.2 Movement and Anatomy/Physiology

**FIGURE 4:**
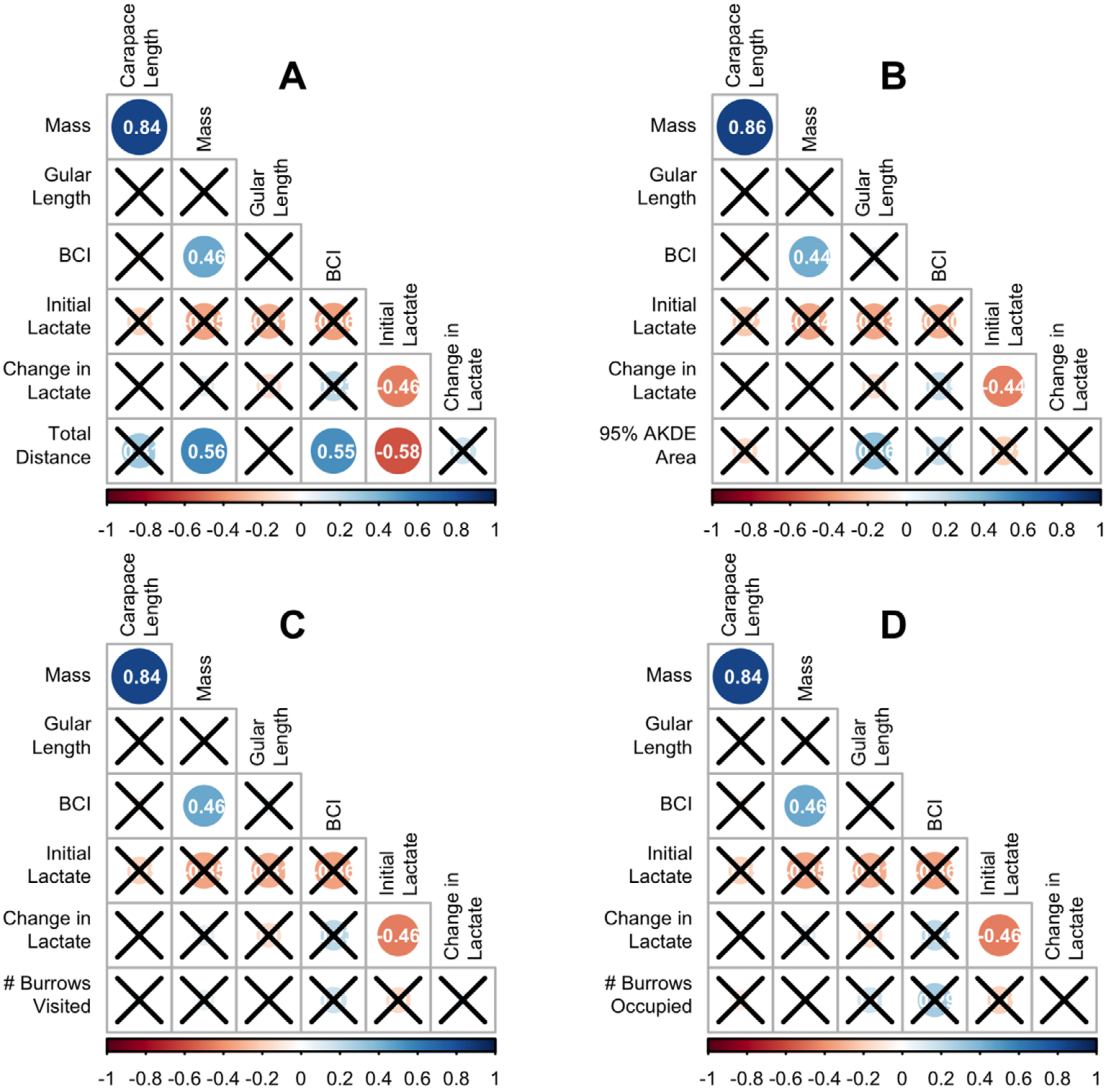
Pearson correlation matrix of the tortoise morphological and physiological traits with total distance traveled (A), 95% AKDE home range area (B), number of burrows visited (C), and number of burrows occupied (D). Circle size, color, and text indicate the correlation between each pair of variables, and an “X” indicates nonsignificant correlations.

**FIGURE 5:**
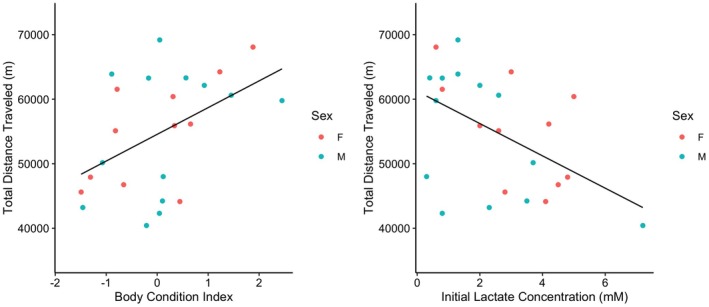
The total distance traveled is plotted versus BCI (left) and initial lactate concentration (right) for each tortoise with the line of best fit in black.

There was a significant linear relationship between total distance traveled and tortoise anatomy and physiology traits as shown in Table 1 and Figure 4. The model with the lowest AICc relating total distance traveled to tortoise anatomy and physiology included only BCI and initial lactate concentration. There was a positive relationship between total distance traveled and BCI, but a negative relationship between total distance traveled and initial lactate concentration, as shown in Figures 4 and 5. While there was a positive correlation between total distance traveled and mass, this is likely because mass is positively correlated to BCI. The residuals of the best model are normal and homoscedastic. There was not a significant linear relationship between 95% AKDE and any tortoise anatomy and physiology traits, nor was there a significant linear relationship between number of burrows visited or occupied and any tortoise anatomy and physiology traits, as shown in Table 2, Figure 4, and Tables S2 and S3.

**TABLE 1:** Linear models relating total distance traveled to *Gopherus polyphemus* anatomy and physiology. The Akaike Information Criterion value, *p*‐value, *F*‐value, and *R* ^2^ of the model are reported along with the results on the coefficient estimate, standard error, *t*‐value, and *p*‐value and ANOVA *F*‐value and *p*‐value results for each predictor variable.

Model				Parameter	Coefficients				ANOVA results	
AICc	*p*	*F*	*R* ^2^		Estimate	Std. error	*t*	*p*	*F*	*p*
523.122	**< 0.001**	9.713	0.469	**(Intercept)**	60060.9	2715.1	22.121	**< 0.001**	—	—
				**BCI**	3507.8	1490.2	2.354	**0.028**	12.508	**0.002**
				**Initial Lactate**	−2221.3	844.5	−2.630	**0.015**	6.919	**0.015**
525.881	**0.002**	11.640	0.336	**(Intercept)**	61977.9	2834.3	21.867	**< 0.001**	—	—
				**Initial Lactate**	−2936.2	862.2	−3.405	**0.002**	11.596	**0.002**
527.102	**0.004**	9.948	0.302	**(Intercept)**	5469.0	1657.0	32.639	**< 0.001**	—	—
				**BCI**	4918.0	1559.0	3.154	**0.004**	9.948	**0.004**
537.757	**0.023**	3.239	0.572	(Intercept)	38065.0	91793.0	0.415	0.684	—	—
				Sex (M)	444.8	5705.1	0.078	0.939	0.362	0.555
				CL	101.5	494.5	0.205	0.840	3.961	0.063
				**BCI**	3657.3	5446.0	0.672	0.511	13.144	**0.002**
				GL	−186.9	260.1	−0.719	0.482	0.284	0.601
				Mass	0.5	10.2	0.053	0.958	0.001	0.974
				**Initial Lactate**	−2541.4	1154.4	−2.201	**0.042**	4.107	0.059
				Change in Lactate	−410.0	454.1	−0.903	0.379	0.815	0.379

*Note:* Bold values indicate statistical significance of the variables.

**TABLE 2:** Linear models relating 95% AKDE area to *Gopherus polyphemus* anatomy and physiology. The Akaike Information Criterion value, *p*‐value, *F*‐value, and *R* ^2^ of the model are reported along with the results on the coefficient estimate, standard error, *t*‐value, and *p*‐value, and ANOVA *F*‐value and *p*‐value results for each predictor variable.

Model				Parameter	Coefficients				ANOVA results	
AICc	*p*	*F*	*R* ^2^		Estimate	Std. error	*t*	*p*	*F*	*p*
40.362	**0.009**	8.262	0.282	**(Intercept)**	0.494	0.164	3.002	**0.007**	—	—
				**Sex (M)**	0.629	0.219	2.874	**0.009**	8.262	**0.009**
44.191	**0.036**	3.478	0.354	(Intercept)	−1.395	1.988	−0.701	0.492	—	—
				**Sex (M)**	0.764	0.259	2.945	**0.008**	8.311	**0.010**
				CL	0.006	0.006	0.946	0.356	0.727	0.404
				BCI	0.123	0.104	1.182	1.397	2.286	0.252
56.699	0.104	2.131	0.499	**(Intercept)**	−13.74	6.41	−2.142	**0.049**	—	—
				**Sex (M)**	1.100	0.401	2.742	**0.015**	8.446	**0.011**
				**CL**	0.078	0.035	2.159	**0.047**	0.739	0.403
				**BCI**	0.840	0.377	2.228	**0.042**	1.420	0.252
				GL	−0.011	0.018	−0.587	0.566	0.176	0.681
				Mass	−0.001	0.001	−1.959	0.069	3.923	0.066
				Initial Lactate	0.035	0.080	0.442	0.665	0.196	0.664
				Change in Lactate	0.004	0.031	0.120	0.906	0.014	0.906

*Note:* Bold values indicate statistical significance of the variables.

### 3.3 Anatomy and Physiology Results

On average, preexercise, baseline lactate was 3.0 (range 0.3–9.7, *n* = 26) mM, and postexercise lactate was 5.9 (range 1.3–17.5, *n* = 25) mM. The average change in lactate was 3.2 (range −4.4 to 14.0, *n* = 25) mM. There was not a significant linear relationship between initial lactate and tortoise anatomy traits, sex, and activity/temperature upon capture as shown in Figure S2 and Table S4. There was not a significant linear relationship between change in lactate concentration and tortoise anatomy traits, sex, activity, or temperature upon capture as shown in Figure S2 and Table S5.

## 4 Discussion

Vertebrate movement patterns depend on physiological and social factors, and this study found that both body condition and lactate physiology are drivers of how *Gopherus polyphemus* balances tradeoffs within and between the sexes. Our hypothesis that Gopher Tortoises with higher body condition and greater aerobic fitness (as assessed by change in lactate due to exercise) would travel more distance, have a larger home range, and use more burrows was partially supported. The values of the different movement metrics were not all correlated to one another, specifically, home range area and burrow use were not correlated to total distance traveled. Change in lactate concentration in response to exercise was not correlated to any movement metrics. However, total distance traveled was positively correlated to BCI and negatively related to initial lactate concentration, suggesting that how far a Gopher Tortoise moves is physiologically constrained. BCI serves as a proxy for the relative extent of an individual's energy reserves, and individuals with higher BCIs are predicted to have larger energy reserves (Stevenson and Woods Jr 2006). Baseline lactate levels are related to turtle health, with elevated levels associated with infection in Eastern Box Turtles ( *Terrapene carolina* ) (Klein et al. 2021) and turtle mortality risk (Gregory et al. 2022; Tucker‐Retter and Lewbart 2022). Gopher Tortoise home range area and burrow use were not correlated to physiology, and so we found no evidence that they were physiologically constrained. In contrast, Stiffler et al. (2024) found a negative correlation between baseline lactate and home range size in the same population of Gopher Tortoises but used more traditional radiotelemetry methods with fewer location observations per individual than this study. The different results between the various movement metrics in this study suggest that home range area and burrow use reflect trade‐offs in movement patterns as limited by the physiologically constrained total distance traveled. Restated, individuals have a finite, movement budget (total distance traveled) that is determined by physiology, and based on their movement strategy can choose, for example, to intensively travel in and occupy a small area and few burrows (e.g., possible territorial behavior) or to cover a larger area but not as intensively occupy any one portion.

A consideration with our change in lactate measurements is that they were based on the same exercise challenge (400 paces). Prior studies using change in lactate as a metric exercised animals to fatigue (such as loss of righting behavior), such that each animal exercised a different amount, but reached the same physiological endpoint. Notably, there is substantial variation in lactate levels at exhaustion (Gardner et al. 2022; Hawthorne and Goessling 2020). Given that Gopher Tortoises can simply close their limbs into their shells and cease moving, we were not able to exercise animals in this study to the point of fatigue, so we standardized the exercise challenge to avoid confounding behavioral responses (i.e., withdrawing limbs into shell) with physiological responses (i.e., exhaustion). However, the standardized exercise in this study did not appear to cause equal aerobic stress among individuals, which likely contributed to the absence of an observed relationship between change in lactate concentration and movement. Additionally, of the 25 tortoises with initial and final lactate concentrations measured, five had lactate levels decrease during exercise. This result could be due to a shift to aerobic metabolism promoting greater efficiency during exercise compared to resting, because turtles respire in part by moving their limbs, and corresponds with other studies that found higher lactate levels turtles with low activity behavior compared to more active behavior (Adamovicz et al. 2018; Klein et al. 2021).

Our hypothesis that male tortoises would travel more distance, have larger home ranges, and use more burrows than female tortoises was partially supported. There was not a significant difference in the total distance traveled and the number of burrows visited between male and female tortoises. However, there was a significant difference in the home range area and the number of burrows occupied between male and female tortoises, with male tortoises having larger home range areas and occupying more burrows. Our result that male tortoises have larger home range areas than females is consistent with many prior studies (Castellón et al. 2018; Eubanks et al. 2003; Guyer et al. 2012; McRae et al. 1981; Metcalf et al. 2023), but some report no difference in home range areas by sex (Guyer et al. 2024; Metcalf et al. 2023). Our result that male tortoises occupy more burrows than females is consistent with prior studies (Boglioli et al. 2003; Eubanks et al. 2003; Guyer et al. 2012; Stiffler et al. 2024).

Our result that there was not a significant difference in total distance traveled between male and female tortoises is in contrast with many prior studies that report male tortoises move more than females based on location data collected at less frequent intervals (Castellón et al. 2018; Douglass and Layne 1978; Eubanks et al. 2003; Guyer et al. 2012; Metcalf et al. 2023). This discrepancy is likely because this study used GPS loggers to collect fine‐scale location data (every 15 min), which include movements that would have been lost at the coarser temporal resolution (1–3 locations per week) of radiotelemetry‐based studies. This consideration is especially pertinent because tortoises spend most of their time around their burrows, so coarse resolution location data typical of telemetry would report no movement if a tortoise was in the same burrow from week to week, missing daily movements around that same burrow. The mean daily distance traveled in this study, 331 m, is much higher compared to a previous estimate of 8.6 m in males and 5.5 m in females (Metcalf et al. 2023) and compared to displacement distances between subsequent (2 days to 1 week) location fixes in telemetry studies, 85 m in males and 54 m in females (Eubanks et al. 2003) and 50–125 m in males during the mating season (Guyer et al. 2012). This is consistent with the results of Stemle et al. (2022) that used GPS loggers on juvenile Gopher Tortoises and reported a much greater daily distance traveled compared to prior literature values derived using telemetry‐based studies. The discrepancy between distance traveled as measured by GPS loggers and measured by radiotelemetry methods is likely true in other taxa. This concurs with the results reviewed by Nathan et al. (2022), that in many vertebrate systems the patterns observed in movement studies depend on the temporal resolution of location fixes.

Despite the fact that male and female *Gopherus polyphemus* moved similar distances, there was a significant difference in home range size between males and females. This paradoxical result, namely different sized home ranges but equal movement (distance traveled) between sexes, is striking given that we would not have been able to measure this important sex effect without autonomous GPS loggers. Specifically, the daily maximum displacement measurements show that female Gopher Tortoises perform more small‐displacing movements within their smaller home range, so their movement levels are likely to be underestimated using temporally coarse scale telemetry data. Thus, movement patterns vary by sex in Gopher Tortoises, suggesting the presence of sex‐based movement strategies. There are possible adaptive explanations for the differences in home range areas by sex despite similar distances traveled. Male Gopher Tortoises performing high‐displacing movements across their large home range would encounter and mate with more females within the context of a scramble‐competition polygamy mating system, thus increasing their evolutionary fitness (Boglioli et al. 2003; Johnson et al. 2009). Recent observations of female–female aggression in Gopher Tortoises in the context of defending nest sites suggest possible territorial behavior in females (Radzio et al. 2017). Thus, moving extensively within a smaller home range may allow female Gopher Tortoises to defend their nest sites and ensure optimal conditions for their eggs, increasing their evolutionary fitness. The fact that we found female tortoises move just as much as males, but occupy much smaller home ranges, forces a conclusion that female movements are not as spatially simple as they have typically been considered across most field‐based ecology studies. Restated, traditional considerations about female animal home ranges have generally ignored that females may be moving the same amount or more than males within their home ranges, as evidenced by our data. These results imply that the general understanding that males move more than females in many taxa based solely on radiotelemetry home range estimates (Aronsson et al. 2016; Giroux et al. 2025; McLoughlin and Ferguson 2000) requires reexamination using methods like GPS loggers that account for total distance traveled to properly consider nuances of space use strategies between the sexes and implications for selection on sexual dimorphism in size and spatial reasoning (Jones et al. 2003; Ofstad et al. 2016).

A limitation with this study is that we only considered physiology at one timepoint. With the increasing availability of biologgers, such as temperature and heart rate monitors, future studies should consider combining GPS loggers and biologgers to connect animal movement to physiology more directly (Hetem et al. 2025). Another limitation is that we had data from 30 tortoises over a 5‐month period; further studies using more tortoises and over a longer time period, including other seasons, should be conducted to determine the generalizability of these results. Gopher Tortoise movement patterns vary with population density (Guyer et al. 2012), so future studies should employ GPS loggers on other Gopher Tortoise populations for comparisons with our results from a high‐density population. A goal with fine‐scale location data is to determine the internal and external factors that determine an animal's path of motion (Nathan et al. 2008). A future direction is to develop criteria to link Gopher Tortoise movement patterns (e.g., distance traveled, velocity, locations visited) with specific behaviors, such as thermoregulation, foraging, socializing, or mating. This would be useful to address questions of how Gopher Tortoises prioritize different activities within their energy and time budgets to identify possible trade‐offs or alternative strategies.

## Author Contributions

**Karin Ebey:** conceptualization (equal), formal analysis (equal), investigation (equal), methodology (equal), writing – original draft (lead), writing – review and editing (equal). **Michael Hilton:** conceptualization (equal), formal analysis (equal), methodology (equal), writing – review and editing (equal). **Jeffrey Goessling:** conceptualization (equal), investigation (equal), methodology (equal), writing – review and editing (equal).

## Funding

This study was funded by the 2024 Gopher Tortoise Council J. Larry Landers Student Research Grant, the Friends of Boyd Hill Nature Preserve, Peter Robison, and the Shell Oil Foundation.

## Conflicts of Interest

The authors declare no conflicts of interest.

## Supporting information


**Table S1:** The ID number, sex, carapace length, mass, gular length, body condition index, initial lactate concentration, final lactate concentration (after the exercise challenge) and change in lactate concentration (as a result of the exercise challenge) is reported for each tortoise.
**Table S2:** Linear models relating number of burrows visited to *Gopherus polyphemus* anatomy and physiology. The Akaike Information Criterion value, *p*‐value, *F*‐value, and *R* ^2^ of the model are reported along with the results on the coefficient estimate, standard error, *t*‐value, and *p*‐value and ANOVA *F*‐value and *p*‐value results for each predictor variable.
**Table S3:** Linear models relating number of burrows occupied to *Gopherus polyphemus* anatomy and physiology. The Akaike Information Criterion value, *p*‐value, *F*‐value, and *R* ^2^ of the model are reported along with the results on the coefficient estimate, standard error, *t*‐value, and *p*‐value and ANOVA *F*‐value and *p*‐value results for each predictor variable.
**Table S4:** Linear models relating initial lactate concentration to tortoise anatomy, physiology, and status on capture. The Akaike Information Criterion value, *p*‐value, *F*‐value, and *R* ^2^ of the model are reported along with the results on the coefficient estimate, standard error, *t*‐value, and *p*‐value and ANOVA *F*‐value and *p*‐value results for each predictor variable.
**Table S5:** Linear models relating change in lactate concentration to tortoise anatomy, physiology, and status on capture. The Akaike Information Criterion value, *p*‐value, *F*‐value, and *R* ^2^ of the model are reported along with the results on the coefficient estimate, standard error, *t*‐value, and *p*‐value and ANOVA *F*‐value and *p*‐value results for each predictor variable.
**Figure S1:** A custom made, hand‐powered treadmill was used for the Gopher Tortoise exercise challenge (walking 400 paces) in this study.
**Figure S2:** Pearson correlation matrix assessing the linear relationship between each pair of tortoise anatomy, physiology, and status on capture with lactate measurements. Circle size, color, and text indicate the correlation between each pair of variables, and an “X” indicates nonsignificant correlations.

## Data Availability

Data and R code are available in a repository titled “Gopher Tortoise Movement; Boyd Hill Nature Preserve 2024‐2025” accessible at https://data.mendeley.com/datasets/8s2vvbstjb/2.
