# Supplementary material for: Mechanisms of Movement Patterns: Physiology and Sex Influence Gopher Tortoise (Gopherus polyphemus) Movement
Source: Ecol Evol. 2026 Jun 30;16(7):e73878. doi: 10.1002/ece3.73878 (PMC13316722; doi:10.1002/ece3.73878)
Supplement: Supplementary file 1 — Table S1: The ID number, sex, carapace length, mass, gular length, body condition index, initial lactate concentration, final lactate concentration (after the exercise challenge) and change in lactate concentration (as a result of the exercise challenge) is reported for each tortoise. Table S2: Linear models relating number of burrows visited to Gopherus polyphemus anatomy and physiology. The Akaike Information Criterion value, p‐value, F‐value, and R 2 of the model are reported along with the results on the coefficient estimate, standard error, t‐value, and p‐value and ANOVA F‐value and p‐value results for each predictor variable. Table S3: Linear models relating number of burrows occupied to Gopherus polyphemus anatomy and physiology. The Akaike Information Criterion value, p‐value, F‐value, and R 2 of the model are reported along with the results on the coefficient estimate, standard error, t‐value, and p‐value and ANOVA F‐value and p‐value results for each predictor variable. Table S4: Linear models relating initial lactate concentration to tortoise anatomy, physiology, and status on capture. The Akaike Information Criterion value, p‐value, F‐value, and R 2 of the model are reported along with the results on the coefficient estimate, standard error, t‐value, and p‐value and ANOVA F‐value and p‐value results for each predictor variable. Table S5: Linear models relating change in lactate concentration to tortoise anatomy, physiology, and status on capture. The Akaike Information Criterion value, p‐value, F‐value, and R 2 of the model are reported along with the results on the coefficient estimate, standard error, t‐value, and p‐value and ANOVA F‐value and p‐value results for each predictor variable. Figure S1: A custom made, hand‐powered treadmill was used for the Gopher Tortoise exercise challenge (walking 400 paces) in this study. Figure S2: Pearson correlation matrix assessing the linear relationship between each pair of tortoise anatomy, physiology, and status [file ECE3-16-e73878-s001.docx]

**Appendix**

Supplementary Table 1. The ID number, sex, carapace length, mass, gular length, body condition index, initial lactate concentration, final lactate concentration (after the exercise challenge) and change in lactate concentration (as a result of the exercise challenge) is reported for each tortoise.

| **Tortoise ID** | **Sex** | **Total distance traveled (m)** | **Average daily distance traveled (m)** | **Average max daily displacement (m)** | **95% AKDE (ha)** | **Number burrows occupied** | **Number burrows visited** |
| --- | --- | --- | --- | --- | --- | --- | --- |
| 104 | F | 45613 | 291 | 39 | 0.18 | 5 | 9 |
| 304 | M | 36025 | 229 | 42 | 0.51 | 13 | 21 |
| 311 | F | 47919 | 305 | 51 | 10.98 | 16 | 30 |
| 313 | F | 44139 | 281 | 39 | 0.61 | 12 | 23 |
| 315 | F | 68074 | 434 | 41 | 0.32 | 11 | 17 |
| 322 | M | 48007 | 306 | 55 | 0.56 | 4 | 5 |
| 323 | F | 46751 | 298 | 33 | 0.23 | 8 | 17 |
| 328 | M | 60614 | 386 | 59 | 1.70 | 27 | 37 |
| 334 | M | 63895 | 407 | 63 | 1.56 | 18 | 33 |
| 336 | F | 55106 | 351 | 29 | 0.13 | 8 | 12 |
| 343 | F | 64239 | 409 | 47 | 0.59 | 11 | 23 |
| 344 | M | 62140 | 396 | 59 | 1.75 | 19 | 30 |
| 346 | M | 69187 | 441 | 56 | 0.95 | 12 | 19 |
| 348 | F | 61539 | 392 | 53 | 1.28 | 20 | 40 |
| 349 | M | 44230 | 282 | 45 | 1.18 | 16 | 19 |
| 352 | F | 55022 | 350 | 42 | 0.23 | 6 | 9 |
| 353 | F | 55892 | 356 | 39 | 0.24 | 8 | 15 |
| 359 | M | 52972 | 337 | 51 | 2.15 | 20 | 25 |
| 364 | M | 59782 | 381 | 55 | 0.65 | 13 | 15 |
| 373 | M | 63272 | 403 | 48 | 0.66 | 17 | 23 |
| 374 | F | 60404 | 385 | 47 | 0.31 | 8 | 15 |
| 393 | M | 43214 | 275 | 41 | 0.63 | 13 | 18 |
| 395 | M | 40425 | 257 | 57 | 1.45 | 19 | 23 |
| 402 | M | 42315 | 270 | 71 | 2.19 | 16 | 31 |
| 444 | F | 56150 | 358 | 47 | 1.04 | 13 | 27 |
| 99 | M | 63301 | 403 | 49 | 1.23 | 22 | 31 |
| UNM-1 | M | 50162 | 320 | 39 | 4.70 | 7 | 9 |
| UNM-2 | F | 25655 | 163 | 35 | 0.51 | 4 | 4 |
| UNM-3 | F | 39142 | 249 | 33 | 0.28 | 7 | 10 |
| UNM-4 | M | 34885 | 222 | 23 | 0.08 | 5 | 11 |

Supplementary Table 2. Linear models relating number of burrows visited to *Gopherus polyphemus* anatomy and physiology. The Akaike Information Criterion value, p-value, F-value, and R^2^ of the model are reported along with the results on the coefficient estimate, standard error, t-value, and p-value and ANOVA F-value and p-value results for each predictor variable.

| Model | | | |  | Coefficients | | | | ANOVA Results | |
| --- | --- | --- | --- | --- | --- | --- | --- | --- | --- | --- |
| AICc | p-value | F-value | R^2^ | Parameter | Estimate | Std. Error | t-value | p-value | F-value | p-value |
| 208.001 | 0.930 | 0.329 | 0.119 | (Intercept) | -126.29 | 125.48 | -1.006 | 0.328 | - | - |
|  |  |  |  | Sex (M) | 4.796 | 7.799 | 0.615 | 0.547 | 0.056 | 0.815 |
|  |  |  |  | CL | 0.818 | 0.676 | 1.209 | 0.243 | 0.174 | 0.682 |
|  |  |  |  | BCI | 9.565 | 7.445 | 1.285 | 0.216 | 0.621 | 0.442 |
|  |  |  |  | GL | -0.139 | 0.356 | -0.391 | 0.700 | 0.048 | 0.829 |
|  |  |  |  | Mass | -0.0016 | 0.014 | -1.154 | 0.265 | 1.341 | 0.263 |
|  |  |  |  | Initial Lactate | -0.342 | 1.578 | -0.217 | 0.831 | 0.014 | 0.906 |
|  |  |  |  | Change in Lactate | -0.137 | 0.621 | -0.221 | 0.828 | 0.049 | 0.828 |

Supplementary Table 3. Linear models relating number of burrows occupied to *Gopherus polyphemus* anatomy and physiology. The Akaike Information Criterion value, p-value, F-value, and R^2^ of the model are reported along with the results on the coefficient estimate, standard error, t-value, and p-value and ANOVA F-value and p-value results for each predictor variable.

| Model | | | |  | Coefficients | | | | ANOVA Results | |
| --- | --- | --- | --- | --- | --- | --- | --- | --- | --- | --- |
| AICc | p-value | F-value | R^2^ | Parameter | Estimate | Std. Error | t-value | p-value | F-value | p-value |
| 162.259 | 0.094 | 3.048 | 0.117 | (Intercept) | 10.909 | 1.692 | 6.446 | <0.001 | - | - |
|  |  |  |  | Sex (M) | 3.948 | 2.262 | 1.746 | 0.094 | 3.048 | 0.094 |
| 177.521 | 0.309 | 1.298 | 0.348 | (Intercept) | -0.011 | 0.682 | -1.644 | 0.119 | - | - |
|  |  |  |  | Sex (M) | 9.022 | 4.239 | 2.128 | **0.048** | 3.052 | 0.099 |
|  |  |  |  | CL | 0.667 | 0.367 | 1.816 | 0.087 | 0.388 | 0.542 |
|  |  |  |  | BCI | 8.112 | 4.047 | 2.005 | 0.061 | 2.334 | 0.145 |
|  |  |  |  | GL | -0.194 | 0193 | -1.006 | 0.329 | 0.634 | 0.437 |
|  |  |  |  | Mass | -0.012 | 0.008 | -1.623 | 0.123 | 2.418 | 0.138 |
|  |  |  |  | Initial Lactate | 0.180 | 0.858 | 0.210 | 0.836 | 0.177 | 0.679 |
|  |  |  |  | Change in Lactate | -0.098 | -0.291 | 0.120 | 0.775 | 0.085 | 0.775 |

Supplementary Table 4. Linear models relating initial lactate concentration to tortoise anatomy, physiology, and status on capture. The Akaike Information Criterion value, p-value, F-value, and R^2^ of the model are reported along with the results on the coefficient estimate, standard error, t-value, and p-value and ANOVA F-value and p-value results for each predictor variable.

| Model | | | |  | Coefficients | | | | ANOVA Results | |
| --- | --- | --- | --- | --- | --- | --- | --- | --- | --- | --- |
| AICc | p-value | F-value | R^2^ | Parameter | Estimate | Std. Error | t-value | p-value | F-value | p-value |
| 119.373 | 0.187 | 1.657 | 0.406 | (Intercept) | 34.604 | 21.030 | 1.645 | 0.118 | **-** | - |
|  |  |  |  | Sex (M) | -1.338 | 1.329 | -1.007 | 0.328 | 1.014 | 0.328 |
|  |  |  |  | CL | -0.110 | 0.112 | -0.988 | 0.337 | 0.999 | 0.331 |
|  |  |  |  | BCI | -1.324 | 1.219 | -1.086 | 0.293 | 3.982 | 0.062 |
|  |  |  |  | GL | -0.007 | 0.060 | -0.124 | 0.903 | 2.437 | 0.137 |
|  |  |  |  | Mass | 0.001 | 0.002 | 0.543 | 0.594 | 0.061 | 0.808 |
|  |  |  |  | Activity code (Out) | -0.573 | 1.038 | -0.552 | 0.588 | 2.482 | 0.134 |
|  |  |  |  | Temp | -0.152 | 0.183 | -0.829 | 0.419 | 0.620 | 0.442 |

Supplementary Table 5. Linear models relating change in lactate concentration to tortoise anatomy, physiology, and status on capture. The Akaike Information Criterion value, p-value, F-value, and R^2^ of the model are reported along with the results on the coefficient estimate, standard error, t-value, and p-value and ANOVA F-value and p-value results for each predictor variable.

| Model | | | |  | Coefficients | | | | ANOVA Results | |
| --- | --- | --- | --- | --- | --- | --- | --- | --- | --- | --- |
| AICc | p-value | F-value | R^2^ | Parameter | Estimate | Std. Error | t-value | p-value | F-value | p-value |
| 146.766 | 0.128 | 2.489 | 0.098 | (Intercept) | 8.394 | 3.423 | 2.452 | 0.022 | **-** | - |
|  |  |  |  | Time walking | -0.012 | 0.008 | -1.578 | 0.128 | 2.489 | 0.128 |
| 147.089 | 0.106 | 2.491 | 0.185 | (Intercept) | 18.561 | 7.424 | 2.500 | 0.020 | **-** | - |
|  |  |  |  | GL | -0.159 | 0.104 | -1.532 | 0.140 | 2.347 | 0.140 |
|  |  |  |  | **Time walking** | -0.017 | 0.008 | -2.100 | **0.048** | 2.635 | 0.119 |
| 163.247 | 0.203 | 1.595 | 0.166 | (Intercept) | -0.403 | 49.633 | -0.008 | 0.994 | **-** | - |
|  |  |  |  | Sex (M) | 0.925 | 2.992 | 0.309 | 0.761 | 0.096 | 0.761 |
|  |  |  |  | CL | 0.170 | 0.251 | 0.677 | 0.508 | 0.423 | 0.525 |
|  |  |  |  | BCI | 2.742 | 2.734 | 1.003 | 0.331 | 3.275 | 0.089 |
|  |  |  |  | **GL** | -0.254 | 0.147 | -1.726 | 0.104 | 4.760 | **0.044** |
|  |  |  |  | Mass | -0.002 | 0.005 | -0.388 | 0.703 | 0.124 | 0.730 |
|  |  |  |  | Activity code (Out) | -1.097 | 2.541 | -0.432 | 0.672 | 0.127 | 0.726 |
|  |  |  |  | Temp | -0.266 | 0.429 | -0.621 | 0.543 | 0.064 | 0.803 |
|  |  |  |  | **Time walking** | -0.033 | 0.012 | -2.836 | **0.012** | 3.891 | 0.066 |


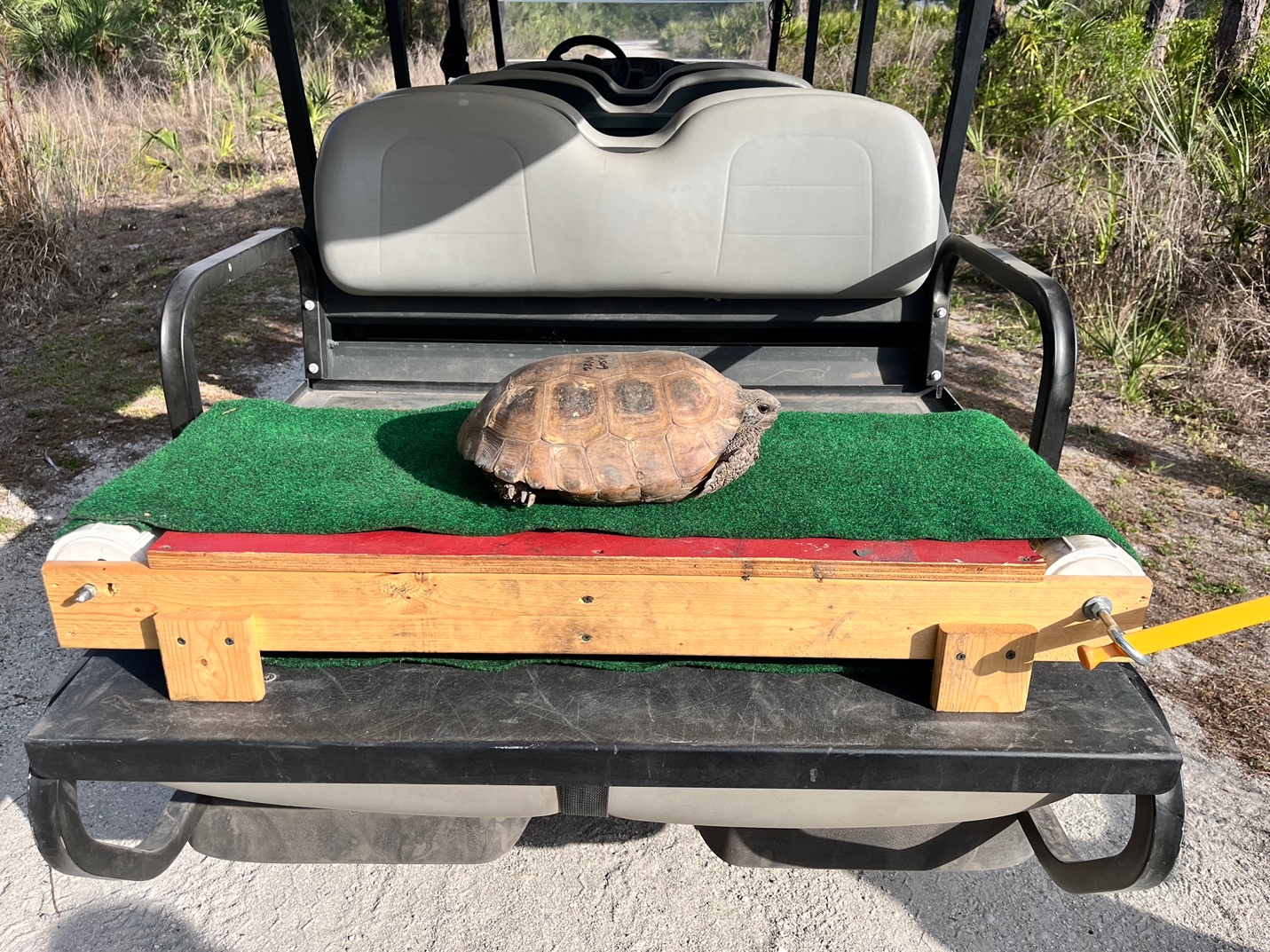


Supplemental Figure 1. A custom made, hand-powered treadmill was used for the Gopher Tortoise exercise challenge (walking 400 paces) in this study.


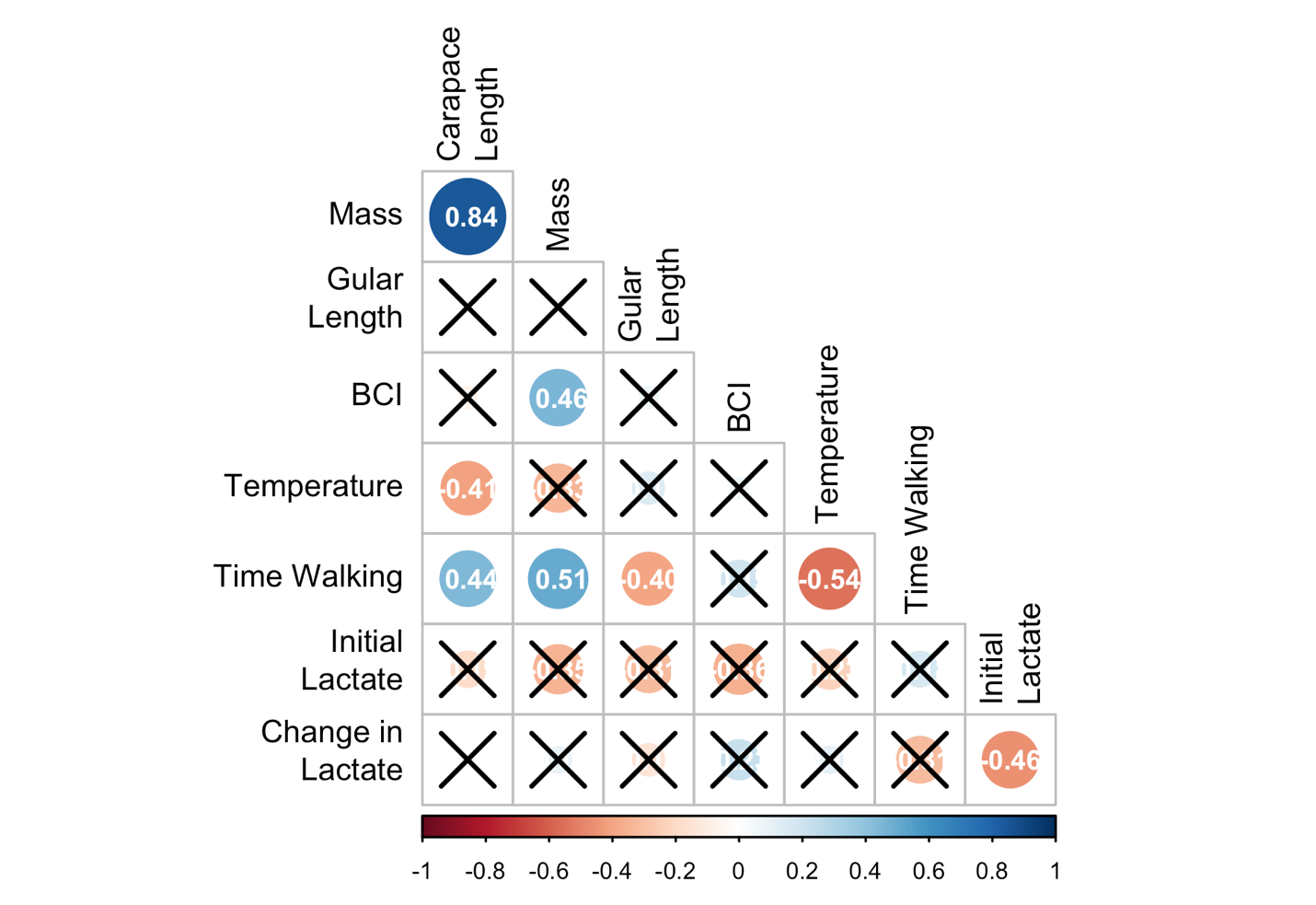


Supplementary Figure 2. Pearson correlation matrix assessing the linear relationship between each pair of tortoise anatomy, physiology, and status on capture with lactate measurements. Circle size, color, and text indicate the correlation between each pair of variables, and an ‘X’ indicates non-significant correlations.
